# A Congo Basin ethnographic analogue of pre-Columbian Amazonian raised fields shows the ephemeral legacy of organic matter management

**DOI:** 10.1038/s41598-020-67467-8

**Published:** 2020-07-02

**Authors:** Leonor Rodrigues, Tobias Sprafke, Carine Bokatola Moyikola, Bernard G. Barthès, Isabelle Bertrand, Marion Comptour, Stéphen Rostain, Joseph Yoka, Doyle McKey

**Affiliations:** 1 Centre d’Écologie Fonctionnelle et Évolutive, CEFE, Univ Montpellier, CNRS, EPHE, IRD, Univ Paul Valéry Montpellier 3, Montpellier, France; 2 Institute of Geography, University of Bern, Hallerstrasse 12, 3012 Bern, Switzerland; 3 Laboratoire de Botanique et Ecologie, Faculté des Sciences, Université Marien Ngouabi, Brazzaville, Congo; 4 Eco&Sols, Université de Montpellier, CIRAD, INRAE, IRD, Montpellier SupAgro, 34060 Montpellier, France; 5 UMR 8096 “Archéologie Des Amériques”, CNRS, Panthéon-Sorbonne University, Maison René Ginouvès, 21 Allée de l’Université, 92023 Nanterre, France; 6 Present Address: 48 Boulevard des Arceaux, 34000 Montpellier, France

**Keywords:** Agroecology, Ecology

## Abstract

The functioning and productivity of pre-Columbian raised fields (RFs) and their role in the development of complex societies in Amazonian savannas remain debated. RF agriculture is conducted today in the Congo Basin, offering an instructive analogue to pre-Columbian RFs in Amazonia. Our study of construction of present-day RFs documents periodic addition of organic matter (OM) during repeated field/fallow cycles. Field investigations of RF profiles supported by spectrophotometry reveal a characteristic stratigraphy. Soil geochemistry indicates that the management of Congo RFs improves soil fertility for a limited time when they are under cultivation, but nutrient availability in fallow RFs differs little from that in uncultivated reference topsoils. Furthermore, examination of soil micromorphology shows that within less than 40 years, bioturbation almost completely removes stratigraphic evidence of repeated OM amendments. If Amazonian RFs were similarly managed, their vestiges would thus be unlikely to show traces of such management centuries after abandonment. These results call into question the hypothesis that the sole purpose of constructing RFs in pre-Columbian Amazonia was drainage.

## Introduction

Recent research in Amazonia has revealed that centuries of human occupation before European arrival have in many sites profoundly altered soils and landscapes^1,2^. The widespread presence of *Terra preta do indio* and similar Anthrosols, collectively termed Amazonian Dark Earths (ADEs)^3,4^, and tree species composition altered by human activities^5–7^, belie previous perceptions of ‘pristine’ Amazonian forests. Seasonally inundated savanna regions of Amazonia offer other striking examples of human-transformed landscapes: large areas of pre-Columbian wetland raised fields (RFs). These are elevated earthen structures of varying size and shape, from round mounds < 2 m in diameter to platforms up to 100 m broad and hundreds of meters long, on which farmers grew flood-intolerant crops^8^. RFs were an important component of subsistence systems for people living in these environments^8,9^. However, soils of RF landscapes have received much less attention than the ADEs that are widespread in forested Amazonia, and how RF soils were managed remains poorly understood^8,10^. Knowledge on the functioning of RF soils is crucial for a better understanding of the societies that depended on them. It could also be useful in conceiving sustainable ways to use similar environments today^11^.

In Amazonia, as elsewhere, one key function of RFs was to provide well-drained ground for crops^8,9,12–14^. But whether making RFs in Amazonian savannas conferred other benefits has been much debated, and the predominant opinions have shifted over time. Early research on Amazonian RFs was inspired by comparisons with the *chinampas*, ancient agricultural systems that have persisted up to the present (although much modified) along lakeshores and perennial wetlands in the valley of Mexico^15,16^. In the *chinampas*, sediments and aquatic vegetation from permanent canals are periodically added to the fields. RF sites in the Ecuadorian highlands may have functioned similarly, and animal manure may also have been applied^17^.

It soon emerged, however, that for sites in Amazonian lowlands such comparisons with the *chinampas* were questionable^12,13,18^. For similar reasons pre-Columbian RFs in the Mesoamerican lowlands also seem not to be close analogues to Amazonian RFs, having been built in pedo-climatic environments very different from those in Amazonia. Like the *chinampas*, RFs in the lowlands of Mexico, Guatemala and Belize (e.g.,^19–21^) were built in perennial wetlands, where Maya farmers adapted to high water tables by piling up marly-gypsic sediments on top of old dark soils. The canals between the RFs are hypothesized to have been used for drainage, for irrigation during dry periods, and to supply sediments and vegetation as nutrient amendments^22^. In contrast, in most Amazonian RF systems, there is no permanent aquatic compartment that could have supplied such resources, and their usually nutrient-poor soils^18–20^ would not have allowed the permanent cultivation that characterizes the *chinampas*, whose soils have both alluvial and volcanic inputs.

Some observations suggest, however, that at least in some Amazonian sites organic amendments were practiced. In the K-VIII site in French Guianan coastal savannas, an area just upslope of RFs has clay-rich subsoil exposed at the surface; its topsoil may have been scraped and deposited on the nearby RFs^23^. In the same site, low fire frequency during the period of RF cultivation suggests fire-free management, in which grass biomass may have been conserved for use as organic amendment though composting^24^. In another French Guianan site, abundance of maize phytoliths in vestiges of RFs suggested accumulation of crop residues that may have been used for a similar purpose^23^. Macroscopic charcoal has only been found at sites where RFs were made in environments that were covered with wetland forest that had to be cleared for their construction, as for example the Maya RFs in Belize^25^ and RFs in one site in the Bolivian lowlands^26^.

Despite these observations, there is no evidence that RF soils in peri-Amazonian savannas have higher amounts of charcoal, soil organic matter (SOM) or nutrients such as phosphorus (P) compared to adjacent reference soils^13,27^. Thus from being viewed as representing a “pre-Columbian green revolution”, Amazonian RFs came to be seen simply as raised planting surfaces to mitigate flood risk^12,26^. Lack of evidence for nutrient amendments has been used to suggest that no organic amendment was practiced^28,29^.

However, there is little doubt that in such highly weathered, acid substrates, nutrient amendment from organic matter would have been crucial for agricultural production, as it is for other nutrient-poor soils throughout the tropics^30^. Organic amendments may have been necessary to assure at least modest yields in an environment with low soil nutrient content. The crops proposed to have been planted on lowland RFs include maize^23^, peanut, squash, sweet potato^31^ and manioc (also known as cassava)^8,14, 18,32^. Archaeobotanical evidence is particularly convincing for maize and squash in RFs of French Guianan coastal savannas^23^. Although manioc can produce yields even on quite poor soils, most of the mentioned crops, including maize, are nutrient-demanding and require fertilization to sustain satisfactory yields in nutrient-poor soils^33^.

In their sole focus on the *chinampas* model, archaeologists have largely ignored the existence of other present-day analogues of pre-Columbian Amazonian RFs that are arguably more pertinent. In several regions in Africa, Asia and Oceania, wetland RF agriculture is practiced today within environmental settings comparable to those in Amazonian savannas where RFs were cultivated in pre-Columbian times. Denevan and Turner, two pioneers in the study of pre-Columbian RFs, pointed to these analogues 45 years ago^34^ but since then few scholars have drawn on them for insights. Although present-day RFs have rarely been studied in detail, descriptions of heavy and localized applications of soil and organic materials are widespread. These practices aim to allow cultivation in flooding areas, but they also increase nutrient concentration in topsoil. They include transfer of topsoil and plant biomass (as organic amendment) from surrounding areas onto RFs^10^. Although the largest present-day RFs are built in wetlands, some are built in non-flooded grassland, where drainage is not a major concern. In such settings, the primary reason for constructing RFs appears to be the improvement of soil physical properties and the enrichment in organic matter that is decomposed and whose nutrients are mobilized to support crop growth^35^.

Seasonal savanna wetlands in the neotropical region—and agricultural systems that were built in them—are subject to the same ecological constraints (i.e. flooding and nutrient-poor soils) as in other parts of the tropics. This suggests that, as in the Old-World tropics, biomass was likely mobilized in some way in pre-Columbian Amazonian RFs to allow cultivation in flooded areas and furnish nutrients to crop plants. Until now, composition of soils in vestiges of these RFs offers no direct evidence that biomass was used (through either decomposition or combustion), as no accumulation of organic carbon or charcoal has been reported^13^. However, we have poor understanding of the ‘signatures’ that are left behind by different agricultural practices and how long these signatures persist^36,37^ . If fertility of RFs was based on added biomass, what traces of such biomass addition could we expect to find in soils and sediments of pre-Columbian RFs? To our knowledge, there exist no studies that explain how construction techniques and management practices affect soil properties of RFs and how these properties change through time, once RFs are abandoned.

**Figure 1:**
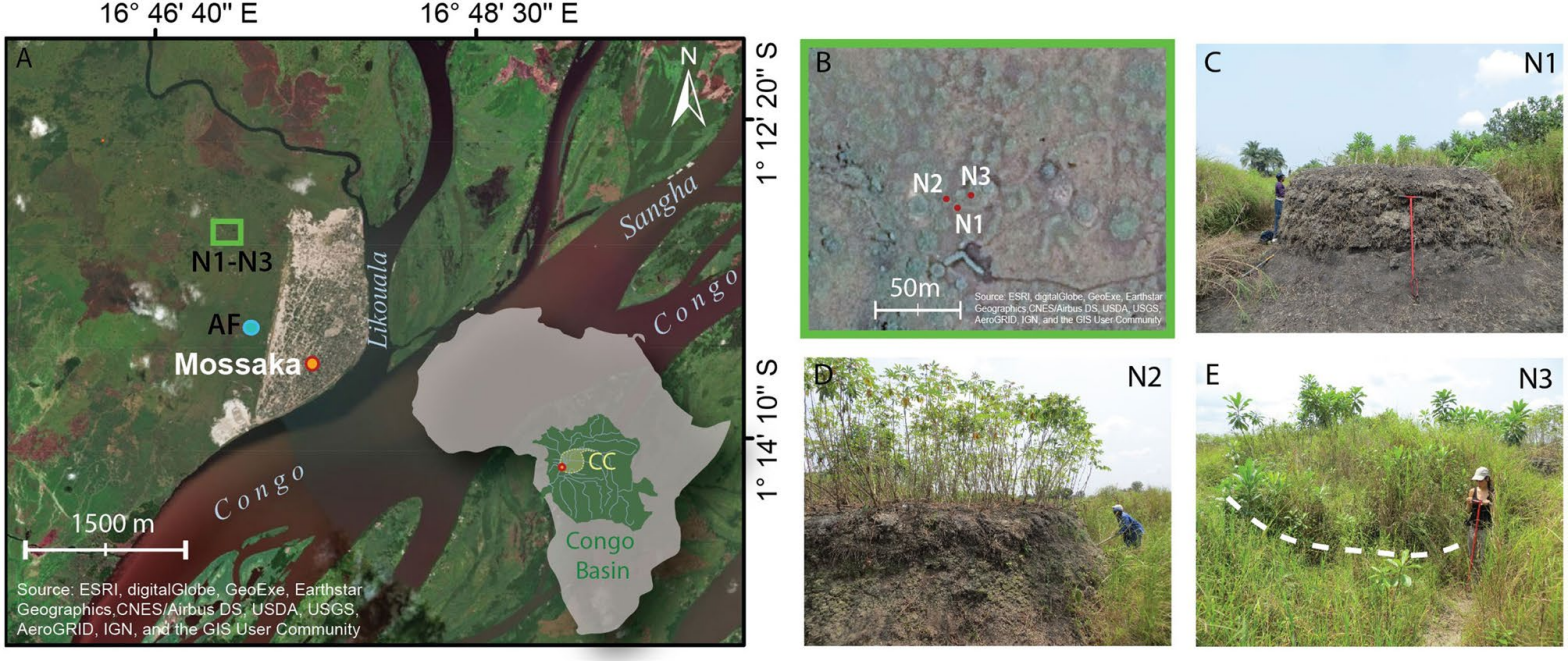
Study area and the four studied fields. **(A)** Study area in the cuvette centrale (CC) of the Congo Basin (satellite imagery from the ESRI ArcGIS base map). Inset map shows the location of the Congo Basin and CC in Africa; red-bordered orange dot (in both inset and main maps) indicates the city of Mossaka (built on sand pumped from the Congo River), green rectangle indicates location of RFs N1–N3 (1°12′59.48"S/ 16°46′56.27"E). Green dot indicates location of the abandoned field AF (1°13′30.52"S/ 16°47′15.71"E). **(B)** Fields N1-N3 shown from the air and **(C–E)** from the ground (satellite imagery from the ESRI ArcGIS base map). In **(E)**, the white dashed line shows the approximate border of the field). Photos (1–20 August 2017), Leonor Rodrigues and Tobias Sprafke.

To overcome this fundamental gap, we studied presently cultivated wetland RFs near Mossaka (Republic of Congo), located in the *cuvette centrale*, the lowest-lying part of the Congo Basin (Fig. 1). The climate, seasonal flooding, and acid, nutrient-poor soils in this region are comparable to those in parts of Amazonia where RFs were cultivated in pre-Columbian times. The agricultural environment in Mossaka also shares an important feature with that of pre-Columbian RF farmers in Amazonia: the absence of domesticated livestock and thus reliance solely on vegetation and SOM as sources of organic amendments. Moreover, manioc, which requires loose and well-drained topsoil and has relatively modest nutrient requirements^38^, is the most important staple crop in both regions, at least today. We provide a detailed description and analysis of how RFs are constructed and maintained today in Mossaka, and how these practices affect the properties of RFs. We characterized soil profiles and their development to determine how topsoil quality varies in space and time, and how soil properties over depth profiles change through time. With this, we aim to understand whether the nutrients concentrated in RFs by material addition persist or accumulate over time, or whether, on the contrary, nutrient concentration in RFs is a fleeting phenomenon.

## Results and discussion

### Construction and maintenance of RFs

**Figure 2:**
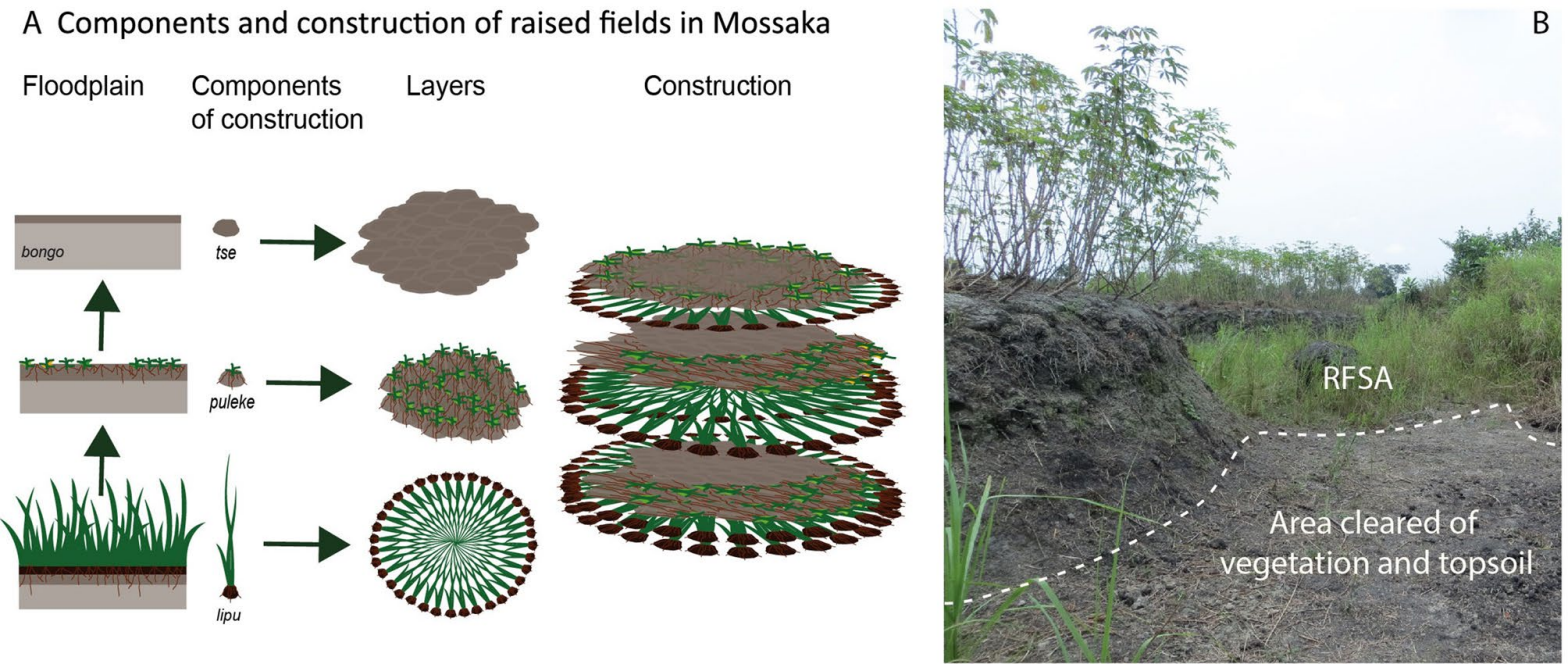
Mode of construction of RFs in Mossaka. **(A)** Materials used to construct RFs, and sequence of placement. *Lipu*: earth clumps with rooted vegetation; *puleke*: topsoil with vegetation debris; *tse:* topsoil from the RFSA; *bongo:* grey clay (C horizon) (Ilustration made with Adobe Illustrator). **(B)** Area cleared of vegetation and topsoil (*tse*) next to field N2 (which was planted with manioc). Photo (1–20 August 2017), Leonor Rodrigues.

Construction of RFs is described in detail by Comptour et al.^11^ and summarized here. RFs in Mossaka are constructed in the floodplain on heavy clay soils where two tributary rivers, the Sangha and the Likouala-Mossaka, join the Congo River (see SI for detailed description of the study area). RFs in Mossaka are permanent structures that, once constructed, are repaired and maintained for decades. They comprise three components, named in the local Likouba language *lipu* (earth clumps along with rooted vegetation), *puleke* (topsoil with vegetation debris) and *tse* (topsoil), which are sequentially piled up on each other (Fig. 2A). RF construction in Mossaka is a (bio-)mass-transfer system: vegetation and soil added to the RF are gathered from the area around the RF, hereinafter termed the RF supply area (RFSA) (Fig. 2B). The total area (RF plus RFSA) usually is about 2.5 times the size of the RF itself.

Vegetation used to construct RFs serves as organic amendment. Earth bound by grass roots also provides physical support, allowing the building of mounds high enough (up to 1.5 m) to protect crops from flooding. RFs are mainly planted with manioc, but sweet potato, pineapple, sugar cane and banana are also grown on RFs. Oil palm trees are generally found on abandoned RFs. Over time, much of the plant biomass added to RFs decomposes and after one or two years of cultivation, fields are left fallow for 5–10 years, depending on the farmer. In this biomass-transfer system, fallow periods serve to regenerate topsoil and vegetation not only on the RF but also and most importantly in the RFSA. The RFSA is the property of the field owner, who alone has the right to collect its topsoil and vegetation. When fallow fields are again put into cultivation, new layers of all three components (*lipu*, *puleke* and *tse*) are collected from the RFSA and added to the hoed RF surface.

### Physical characteristics of soil profiles in RFs

**Figure 3:**
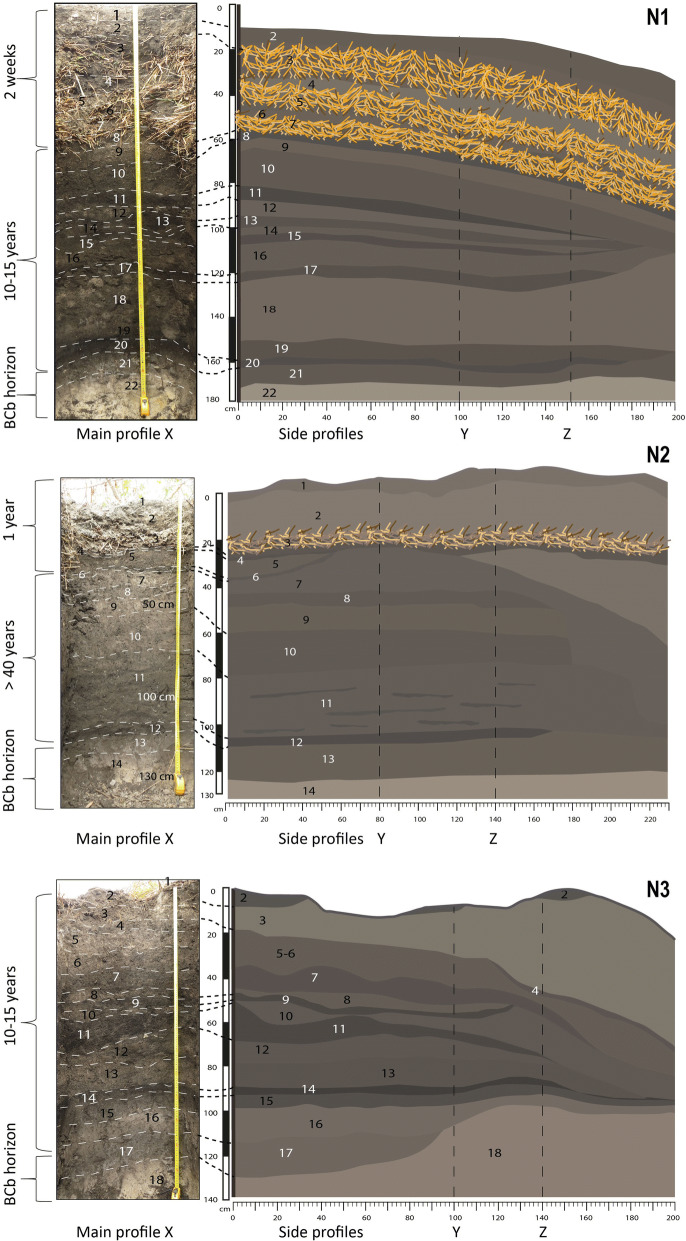
Age and internal morphology of RFs N1–N3. Layers are labeled with numbers. Left panel for each field presents the main profile (X), right panel the two side profiles (Y and Z). Note: layer 1 in side profiles of RF N3 is missing. It was unintentionally removed during our observation and sampling campaign when the field was cleared of fallow vegetation before excavation of the trench. Photos (1–20 August 2017), Leonor Rodrigues and Tobias Sprafke. (Ilustrations made with Adobe Illustrator).

**Figure 4:**
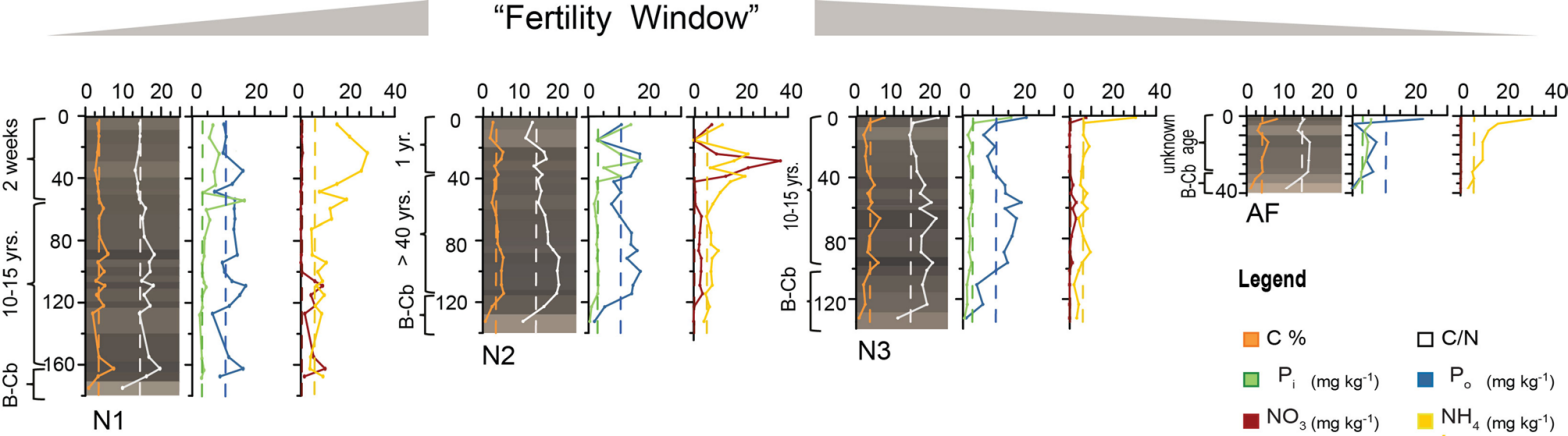
Fertility over time. The three different phases in the cycle of cultivation represented by fields N1 (two weeks after addition of fresh biomass to rehabilitate a field that had been left fallow for at least 10 years), N2 (one year after rehabilitation, field age > 40 years), and N3 (fallow, since about 10 years). Some fields are not only left fallow, but abandoned (AF, abandoned at least 20 years ago). Peak of chemical fertility in the layers where crop roots are concentrated is reached some months to one year after rehabilitation for cultivation (“Fertility Window”). Fertility then declines, returning to values typical of nearby uncultivated soils during the fallow period and falling even lower with abandonment. Plots show downprofile colour (visualized by conversion of spectrophotometrically measured colour space values into the RGB colour space^39^ and chemical analysis of the main profiles (X) of fields N1, N2, and N3. Dashed lines in the background are the mean values for each chemical parameter of the topsoil (Ah) samples taken at 10 cm depth from areas near each field in the RFSA. (Graphs were plotted with Grapher and arranged using Adobe Illustrator).

Soil profiles of four RFs (N1–N3 and AF) located in the floodplain of Mossaka (Fig. 1) were studied. These four fields vary in age and represent different phases in the cycle of cultivation (Figs. 3,4). Fields N1 and N2 were actively cultivated, whereas fields N3 (fallow) and AF (abandoned; Fig. 4) were not. The profiles reveal how the mode of construction and maintenance of RFs influences soil vertical organization, and how this organization changes over time. Field N1 was constructed 15–20 years ago and had been left fallow for at least 10 years just until two weeks before our study, when it was rehabilitated after addition of 50 cm of topsoil and vegetation from the RF and RFSA. Field N2, first constructed more than 40 years ago, had been in fallow for about nine years when it was rehabilitated in 2016 and planted with manioc. This field was thus in its second year of cultivation when the trench was opened (tubers are harvested one to two years after planting). Field N3, built 15–20 years ago, was in fallow when we opened the trench, and had lain fallow for about 10 years. The age of field AF is unknown, but according to local inhabitants, it has been abandoned for more than 20 years. It is thus older than fields N1 and N3.

As a consequence of the mode of construction, near-surface portions of RF profiles show a sequence of alternating layers (grass/topsoil/grass), conspicuous in the upper 50 cm of N1 (Fig. 3A) and still visible in the upper 30 cm of N2 (Fig. 3B), where layers were applied one year before our study. Within two years after construction, the grass layers are largely decomposed. Below the most recently added layer of vegetation, this results in a characteristic stratigraphy, with dark crumbly layers rich in OM alternating with lighter-coloured clay-rich layers relatively poor in OM. In the oldest fields (N2 and AF), this stratigraphy is much less visible, layer boundaries being much more diffuse than in profiles of fields N1 and N3 (Figs. 3,4). Colour measurements by diffuse reflectance spectroscopy visualized as RGB values in Fig. 4^39^ quantify these field observations and reveal only very weak stratigraphy for N2 and AF. In addition, colour data predict the soil carbon content: across all samples (n = 59), the Lightness value L is negatively correlated with soil organic carbon (C_org_) content (R = -0.70, p = 3.3 e−11; Fig. S1, Table S1). The relationship appears to be asymptotic: beyond a certain C_org_ amount the samples cannot get any blacker (Fig. S1 and Table S1). A well-defined 5–10 cm-thick, black, OM-rich layer with sharp boundaries developed at the bottom of each RF (Figs. 3,4). It resulted from decomposition of the first layer of vegetation deposited during the field’s initial construction (first circle of *lipu*, i.e. rooted vegetation with earth clumps; Fig. 2). Beneath this layer occurred the natural BCb horizon, composed of yellow-white clay (Fig. 3). The ancient topsoil (Ahb) of the floodplain soils is missing at the base of each RF, as it was removed and incorporated into the first layer of *lipu*.

### Chemical characteristics and structure of soil profiles

The clay-dominated sediments of the floodplain are extremely weathered, containing almost no bases and consisting largely of silica (mean 59%) and aluminum (mean 20%), resulting in a very high CIA (chemical index of alteration^40^ of 95 (Table S2)). These results are consistent with the measured geochemistry of rivers of the Congo Basin, which are among the most acidic rivers of the world, with low solute concentrations and low amounts of suspended sediments, mainly kaolinite^41^. Thus, as expected, pH values of all soil samples were very low (mean 3.58, standard deviation ± 0.09). In all samples, C_org_ content was generally high (mean value 36 g kg^−1^), ranging from 7.5 g kg^−1^ in the OM-poor pale clay layers at the bottom of the profiles up to 90 g kg^−1^ in some OM-rich black layers (main profiles: Fig. 4 and Table S3; all profiles: Table S3). Values of C/N were generally high (mean = 16) and became slightly larger towards the bottom (max = 21.8) of the profile in all fields, pointing to hampered C mineralization and possible biomass-derived N leaching during the anoxic conditions that occur during annual flooding. The reference samples taken from areas adjacent to RFs had minimum and maximum C_org_ values of 27 g kg^−1^ and 48 g kg^−1^, respectively. The oldest field (N2) had slightly lower C_org_ values than the three other fields and the differences in values between layers were much smaller (Fig. 4; Table S3).

**Figure 5:**
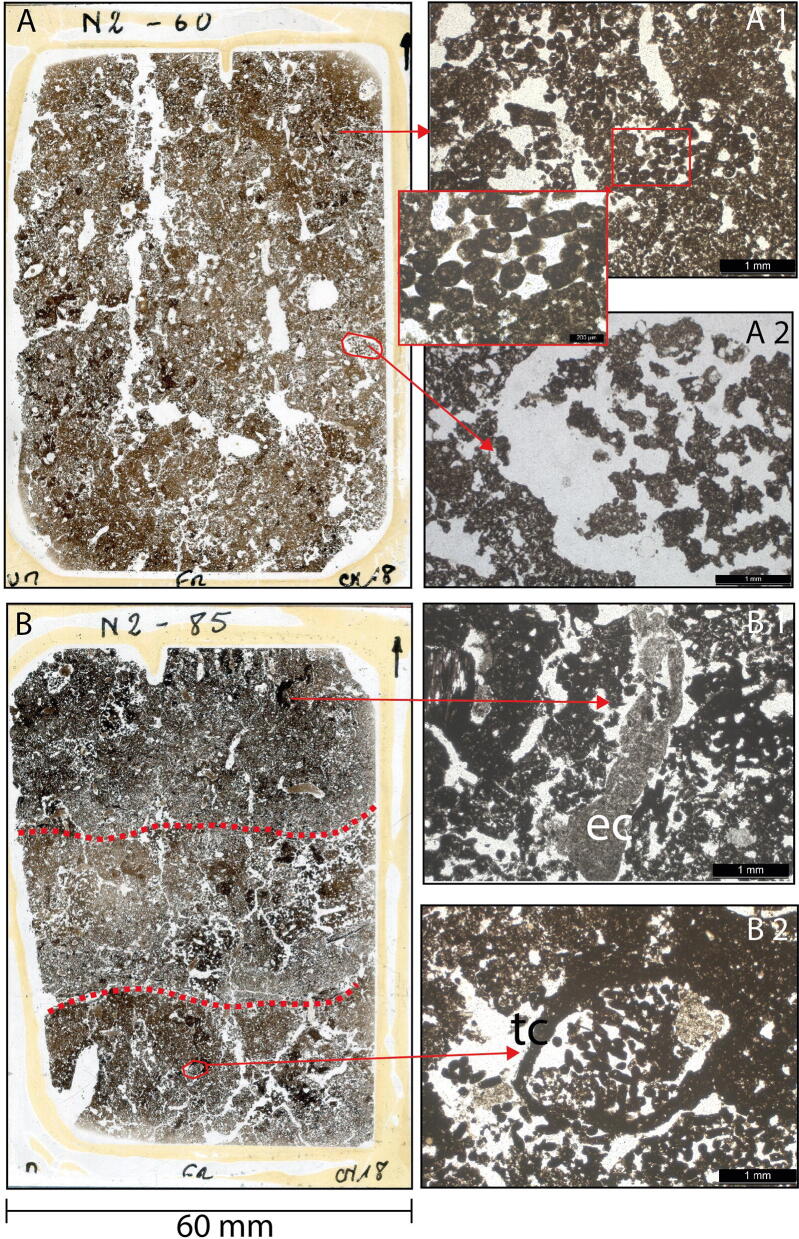
Scans and photomicrographs [plain polarized light (PPL)] from thin sections of field N2 illustrating the alteration of RF soils by biota. **(A)** Thin section from 60 cm depth, a mixed organo-mineral layer with large bio-pores and root channels infilled with faunal excrements. **(A1)** Soil structure made from bacillo-cylindric faunal excrements. **(A2)** Soil pore filled with degraded faunal excrements. B: Thin section from 85 cm depth encompassing three distinct layers (organic-mineral-organic) with boundaries (dashed red lines) blurred by mixing. **(B1)** Earthworm excrements (ec). **(B2)** Channel built by termites/ants (tc) infilled with bacillo-cylindric faunal excrements, most probably produced by small annelids (Enchytraeidae) or mites. Thin sections were analyzed with a Leica DMLP 2000 microscope. For complete documentation of micromorphology, see SI.

Average total phosphorus (P) content from clay underlying the fields was 400 mg P kg^−1^ (Table S2), which is moderate compared to usual global ranges in unmanaged soils (< 100—1,000 mg P kg^−1^) ^42^. Usually, in acidic soils less than 1% of total P is available to plants, as P is bound to elements such as Al and Fe in insoluble complexes^43^. Strikingly, we measured relatively high values of total extractable phosphorus P_tex_ (sum of organic phosphorus (P_o_) and inorganic phosphorus (P_i_)) in the OM-rich layers of the RFs (mean 16.0 mg kg^−1^, max. 49.4 mg kg^−1^) and the reference soils (mean 10.3 mg kg^−1^, max 21.9 mg kg^−1^) (Fig. 5 and Table S3). Given the absence of chemical fertilizer applications and low atmospheric input^42^, local biochemical processes seem to make P available. Assimilation of P from local sediment by grasses adapted to anaerobic conditions is likely possible during the flooding period, when P is released upon reduction of Fe^3+^ to Fe^2+^^44^. Crops require inorganic P (P_i_) ^45^, which shows a striking predominance in the upper part of RFs N1-N3, whereas in the lower parts of these fields, in the abandoned field (AF), and in the reference soils P_o_ predominates. P_i_ becomes available several months to a few years after organic amendments, as maximum contents are in recent former surfaces (which were in fallow before the application of the new material) in N1 (main profile X 50–57 cm) and N2 (profile X 28–29 cm; 37–40 cm). In N2, these layers with high P_i_ values derive from vegetation applied one year before our observations and sample collection. In the fallow field N3, high P_i_ values are restricted to a thin surface layer (main profile X 0–5 cm). In the abandoned field (AF) Pi values are low throughout the profile, even in the surface layer.

The results for mineral nitrogen (N) follow a similar pattern. Mineralization under the very acid conditions observed in our study site leads to high ammonium (NH_4_^+^) values in the upper layers of the fields (Table S3). Nitrification, like P availability, is normally hampered in low-pH milieus, and values of nitrate (NO_3_^-^) are very low throughout profiles of fields N1, N3, and AF. However, nitrate is present in relatively high amounts in field N2, but only in the uppermost actively decomposing layers of the field (highest value 37.3 mg kg^−1^, found at 30 cm). Recent studies demonstrate that nitrification can occur in very acid soils when sufficient substrate is present^46^. The high microbial activity in the uppermost layers of the field could induce release of both nitrate and P_i_ from organic forms remaining in the biomass. Organic matter, crop and microorganisms must interact in some way to produce these high values of P_i_ and NO_3_^-^. Whatever the mechanisms involved, they appear to come into play only after considerable mineralization of OM has occurred, i.e., one year of cultivation of the fields (cf. values for the upper layers of field N2 with those of field N1, to which vegetation was added only recently).

### Soil fauna, bioturbation and micromorphology

Field and microscopic observations indicate the abundance of meso- and macrofauna in the studied RFs. Fallow fields (including field N3) frequently harbor colonies of social insects (termites and ants; see SI for detailed information). Profiles of RF soils and examination of soil micromorphology in thin sections (Fig. 5) show the importance of biota in making RF soils more favorable for crop growth. The method of construction of RFs, with relatively loose vegetation layers sandwiched between topsoil layers, creates a porous structure, and porosity is further increased by activity of soil biota, such as extensive channel building by earthworms and plant roots (see SI Fig. S2 and S3, and Table S4). RF soil is crumbly in structure, with abundant biogenic aggregates. In the upper layers of RF profiles, pores accounted for up to 26% of the surface area of thin sections. This was more than three times the value found in the bottom horizons (7%), which correspond to the ancient natural soil horizon (BCb) (Table S4). This enhanced soil structure is particularly important for the growing of root crops such as manioc, whose tuberous roots must displace soil in order to grow.

Most remarkably, soils of Mossaka RFs are completely reworked by soil mesofauna. Thin sections from OM-rich layers (Fig. 5) show abundant dense concentrations of fecal pellets of unidentified soil mesofauna (probably mites, enchytraeids or collembola). Together, soil macro- and mesofauna and plant roots create new habitats for microbiota, disperse them with their excrements (macrofauna) and facilitate microbial decomposition of organic matter by exudation (roots) and by shredding organic residues before mineralization (macro- and mesofauna)^47^. Amendments with plant biomass and topsoil, and the decomposition of the added organic matter by abundant and intensively active soil biota, thus appear crucial to the functioning, the fertility and the agronomic performance of Mossaka RFs.

### Fate of RF soil profiles over time

The mode of construction and maintenance of RFs produces a characteristic stratigraphy of soil profiles. However, as shown for the two oldest fields, N2 and AF (Fig. 3), most stratigraphic features are quickly lost, and with them the information they provide about soil management practices. The cause is the decomposition and mineralization of the added OM and the intense bioturbation by soil fauna that mixes layers, homogenizing the profile to a depth of at least 100 cm, as seen in the profile X of N2. Below this depth, mineralization and bioturbation are both hampered by the prolonged anoxic conditions due to flooding, as shown by high C/N values and the decreasing amount of biogenic features in thin sections taken from these layers. This leads to preservation of OM from the initial layer of vegetation deposited during field construction, marking the boundary between natural and anthropogenic soil layers.

Many authors have noted the negative impact of bioturbation on the stratigraphic integrity of archaeological sites^48^. Bioturbation and decomposition both occur rapidly in lowland tropical environments when drainage is adequate and soils are well-aerated. The investigated fields show clearly how rapidly this occurs. Within 40 years, stratigraphy in Mossaka RFs is almost completely lost and with it the history of construction, fallow periods and reuse of RFs. Other important factors contributing to the loss of information are the compaction and erosion of the fields after abandonment. RFs in Mossaka are normally built high enough to escape seasonal flooding. Both compaction of fields, because of mineralization of the organic material and reduced macroporosity of the remaining soil, and erosion, due to heavy rains and flooding, might be responsible for the reduction in height of RFs after abandonment. The height of fields varies according to the local flooding height^11^. AF is currently only 0.4 m high. By comparing with fields under cultivation next to AF, which are all about 1–1.3 m in height, we estimate that at least 0.5 m of AF has been lost, to erosion of the upper layers of AF, to compaction, or both. Erosion and compaction have also been observed for pre-Columbian RFs^26^.

These results suggest that if pre-Columbian RFs were constructed and managed in a similar way, these traces would have long since disappeared in the centuries since abandonment. The only stratigraphic trace of management practices observed in Mossaka RFs that might plausibly be expected to persist for centuries (if pedological conditions stay the same) is the OM-rich vestige of the initial layer of vegetation found in the frequently waterlogged soils at the very bottom of present-day RFs, a layer that marks the time of construction.

### No accumulation over time of nutrients added to RFs

Other potential signatures of past organic matter amendment practices might be expected to be more persistent than stratigraphy. Long-term human use of soils, including cultivation, can result in enrichment in certain elements, particularly P^49^, which under acidic conditions is normally stabilized in soil by the formation of insoluble complexes with Fe and Al. Thus P is expected to accumulate in soils amended with organic material^49^ and is often used as a signature of past human land use. The high levels of P_tex_ in the uppermost OM-rich layers of Mossaka RF (containing large amounts of partially decomposed grasses) show that the two main grasses used to construct RFs, *likinga* (*Phacelurus gabonensis* [Steud.] Clayton,) and *litsie* (*Hyparrhenia diplandra* [Hack.] Stapf)^11^, must be efficient in obtaining P from the mineral soil. Despite these repeated inputs, there is no clear accumulation of P in RF soils. The similar absence of P accumulation in vestiges of Amazonian pre-Columbian RFs does not necessarily indicate the absence of organic amendment practices. First, cultivation can lead to depletion of nutrients in soils (including P), not only by decomposition and erosion but also (perhaps even primarily) as a result of removal with harvested crops. Second, depletion of P is particularly likely where OM applied to fields is exclusively of plant origin, as in Mossaka, because plant biomass contains much lower P concentrations than animal manure^44^. Thus, any accumulation of P is likely to be modest and counter-balanced by removal through crop harvest. Agricultural systems without livestock and their manure and without mineral fertilizers are generally characterized by P-mining conditions^50^. The ability to mine P should be particularly great for plants adapted to grow in redoximorphic soils (such as in seasonally flooded savannas), where P is more easily mobilized^44^. As in some other studies^36,49^, our results thus suggest that neither C_org_ nor P are notably accumulated over time in this system, as the values in the oldest fields N2 and AF (with the exception of C_org_ at the base of RFs) are not considerably higher than those in the reference sites.

Chemical fertility is increased during field cultivation, but rapidly declines in fallow fields (Fig. 4) and is even lower in abandoned fields (e.g. P_o_) (Fig. 4). After one year of cultivation both P_i_ and NO_3_^-^ (the most important nutrients for crops) occur in high concentrations, while in the fallow field N3 (except for the uppermost 1 cm) and the abandoned field AF, values decrease to levels comparable to those in the reference soils (Fig. 4) or even lower (P_o_ in AF). The application of biomass and the resulting activities of soil animals that are important for the increased fertility of RFs also have another effect: over time, they largely efface the evidence of the organic matter amendments on which their activity and abundance, and the fertility of RFs, depend.

### Lessons for Amazonian pre-Columbian RFs from this ethnographic analogue

Our characterization of soil profiles of present-day RFs in Africa yields insight into how humans transform soil properties, and how soil properties over depth profiles are transformed through time. The understanding of these “short-term” dynamics is essential for interpreting how soils of similar agricultural systems of the past would be transformed over long periods of time, i.e., centuries or millennia. Our results clearly show that the construction and management of RFs, in combination with bioturbation, result in considerable improvement of soil properties when fields are cultivated compared to the unmanaged natural soils in the surroundings. Our analysis of soil micromorphology shows that strong bioturbation occurs within cultivated RFs, which on the one hand enhances soil structure and nutrient availability, but on the other hand also erases the initial stratigraphy resulting from the field’s initial construction. In the long term, this leads to the loss of information on how fields were constructed and how they functioned when they were cultivated, hampering the interpretation of ancient agricultural systems. Such stratigraphic information may be preserved only under special circumstances, for example, when RFs are buried by fluvial sedimentation, as were Maya RFs in Belize^25^.

The same features as found in Mossaka—periodic flooding, low nutrient contents of highly weathered soils, grasses adapted to redoximorphic soils where P is more soluble, stockless agricultural systems with no animal manure—characterized the natural and agricultural environment of pre-Columbian RFs in Amazonian savannas^9,24, 27–29,51^. So far, soil composition offers no evidence for any organic amendment practices on pre-Columbian RFs: values for charcoal, SOM and P are not higher on RFs compared to adjacent reference soils^13,27^. This mirrors our results in Mossaka, where nutrient availability in the fallow field (N3) and the abandoned field (AF) differs little from that in reference topsoils. If only abandoned or fallow RFs had been studied in Mossaka, this would have resulted in great underestimation of their agronomic potential. In contrast, nutrient availability is much higher in the cultivated RFs. Our results suggest that when pre-Columbian RFs were under cultivation, addition of vegetation (with adherent topsoil) as organic amendment could have conferred great agronomic benefits. This practice would not have resulted in accumulation of OM and nutrients in RF soils. As with stratigraphy, evidence of nutrient enrichment would have been erased by the very functioning of the soil system. Absence of evidence for nutrient accumulation thus cannot necessarily be taken to indicate the absence of organic amendment practices.

No present-day subsistence system provides a perfect analogue of a past system. Environments vary; environments influence, but do not determine, cultural adaptations; and human agency allows different solutions to similar problems^52^. Concerning RF agriculture, as with other kinds of cultural niche construction in seasonally flooded savannas, pre-Columbian systems in Amazonia may resemble present-day African systems in general^53^ but also differ from them in important aspects^54^. Within Africa, how RF agriculture is conducted shows important differences between Mossaka and another recently studied site, in the Bangweulu Basin in Zambia^10,55^. How pre-Columbian Amazonian RFs functioned is likely to show similar variation among sites. Nevertheless, our results from Mossaka show that the conclusion that nutrient-management practices were unimportant in pre-Columbian RFs^28, 29^ is at the very least premature. Our study joins the few that discuss a fundamental problem in geo-archaeology—to what degree can traces of ancient agricultural practices be stored in soils?^37^. Our results underline the importance of comparison with present-day analogues in interpreting past agricultural systems and suggest promising lines for future geo-archaeological investigations.

## Materials and methods

### Study area

The studied RFs are located in a seasonal floodplain in the Congo Basin’s *cuvette centrale*, near the city of Mossaka, located on the Congo River near its confluence with two tributaries (Fig. 1). Floodplain soils are predominantly heavy clays. Negligible quantities of sediments are deposited during flooding, which occurs during the more intense of two rainy seasons (September–November). RFs are constructed to be high enough to prevent flooding. RF agriculture was once much more widespread than today in the cuvette centrale (see SI text for details).

### Studied fields and sample collection

Fields N1-N3 were round, varying from 4–7.5 m in diameter and from 1.3–1.7 m in height. In each field we opened a trench (up to 4 m broad and 7 m long in the largest field) from the top to the base of the field. We described profiles and identified soil horizons and layers following the guidelines for soil description of the FAO^56^. Following trench opening, we sampled soil from each layer, when these were visible, or at 5-cm depth intervals (total of 220 samples). In each trench, three profiles (X, Y, Z) were sampled (Fig. 3). Ah horizons (0–10 cm depth) were sampled with an auger in seven reference sites for comparison of their properties with those of soils from the RFs. Reference sites were chosen as close as possible to RFs (to minimize natural variation in soil properties) but avoiding RFSAs used to supply biomass and topsoil for building RFs. We also sampled and studied a ridge field (AF) that had long been abandoned (> 20 years), to gain additional information on soil processes though time. This field was 0.4 m in height, 13 m long and 2 m wide. To better understand bioturbation, 14 separate undisturbed blocks of soils (~ 12 cm high/10 cm wide/4 cm thick) were collected for preparation of thin sections for micromorphological analysis.

### Physico-chemical analyses

Soil colour was measured spectrophotometrically. Total elemental composition was determined by X-ray fluorescence spectroscopy (XRF) for six samples (two replicates in each field) representing the C horizon beneath the RFs. The weathering index was calculated using the Chemical Index of Alteration (CIA = 100 × Al_2_O_3_/(Al_2_O_3_ + CaO + Na_2_O + K_2_O) of Nesbitt and Young^40^; pH was measured in KCl. Soil extractable mineral and organic P forms were determined following Olsen^57^ and Ohno and Zibilske^58^. NO_3_^−^ and NH_4_^+^ were determined by continuous flow colorimetry after extraction with KCl. Total C and N concentrations were analyzed by dry combustion and gas chromatographic separation. Carbonates were absent; all C was considered organic (C_org_). Analyses were performed on 194 samples with the exception of colour and pH, for which only the main profiles X (Fig. 3) were analyzed (59 samples), and of total elemental composition of the clays, for which just one sample per field was measured. Details of the physico-chemical analyses are provided in the SI.

### Micromorphology

Fourteen blocks of undisturbed soil taken from fields N2 and N3 (profile X) were saturated with 100% ethanol and then gradually impregnated in vacuum by capillarity with a two-component epoxy resin (Araldite 2020). Thin sections prepared from OM-rich and OM-poor layers taken from different soil depths were analyzed following guidelines of Stoops et al.^59^ and of Bullock et al.^60^ for description of excremental pedofeatures. Details are provided in the SI.

## Supplementary information

Supplementary information 1
