# Supplementary material for: A Congo Basin ethnographic analogue of pre-Columbian Amazonian raised fields shows the ephemeral legacy of organic matter management
Source: Sci Rep. 2020 Jul 2;10:10851. doi: 10.1038/s41598-020-67467-8 (PMC7331663; doi:10.1038/s41598-020-67467-8)
Supplement: Supplementary file 1 — Supplementary information 1 [file 41598_2020_67467_MOESM1_ESM.pdf]

Title: A Congo Basin ethnographic analogue of pre-Columbian Amazonian raised fields shows the ephemeral legacy of organic matter management

Leonor Rodrigues[1,2], Tobias Sprafke[2], Carine Bokatola Moyikola[3], Bernard G. Barthès[4], Isabelle Bertrand[4], Marion Comptour[1,5], Stéphen Rostain[6], Joseph Yoka[3], Doyle McKey[1]

[1]Centre d'Écologie Fonctionnelle et Évolutive, CEFE, CNRS, University of Montpellier, University Paul Valéry Montpellier 3, EPHE, IRD, 1919 Route de Mende, 34293 Montpellier 5, France.
[2]Institute of Geography, University of Bern, Hallerstrasse 12, 3012 Bern, Switzerland.
[3]Université Marien Ngouabi, Laboratoire de Botanique et Ecologie, Faculté des Sciences, Brazzaville, Congo.
[4]Eco&Sols, Université de Montpellier, CIRAD, INRAE, IRD, Montpellier SupAgro, 34060 Montpellier, France.
[5]Current address: 48 Boulevard des Arceaux, 34 000 Montpellier, France.
[6]UMR 8096 "Archéologie des Amériques" CNRS, Panthéon-Sorbonne University, Maison René Ginouvès, 21 allée de l'Université, Nanterre 92023, France.

Corresponding author: Leonor Rodrigues, Institute of Geography, University of Bern, Hallerstrasse 12, 3012 Bern, Switzerland. Telephone : +41 784085352, e-mail address: leonor.rodrigues@giub.unibe.ch

SI Text

Study area. The studied RFs are located in a savanna floodplain near the city of Mossaka, Republic of Congo, in the *cuvette centrale* of the Congo Basin and close to the Congo River (Fig. 1). Mossaka is a small but fast-growing city, counting 15,000 inhabitants (1981: 6,000) [1,2]. The *cuvette centrale* is an intracratonic basin filled with Quaternary sediments resulting in an almost flat area (mean altitude: 230 m a.s.l.) crossed by a major river, the Congo, and its tributaries. At the confluence of the Congo River and several of its major tributaries, the *cuvette centrale* is home to extensive wetlands covering an approximate total area of 190,000 km^2, of which 69,000 km^2 lies in the Republic of Congo [3]. The region's climate, subject to the Atlantic monsoon system, is characterized by two rainy seasons (September-October and March-May) and two dry seasons (July-August and December-February) each year [4]. Compared to the Amazon Basin, the hydrology of the Congo Basin is very little explored. Water in wetlands of the *cuvette centrale* derives more from rainfall and runoff from nearby uplands than from river transport. Fluctuations in water levels are mainly controlled by changes in surface water storage, much more than by changes in underground storage [5]. Detailed ecological studies, including investigations of soil properties and vegetation composition and dynamics, are also scarce. Diverse soils, from Arenosols to clay-rich hydromorphic Gleysols, have been reported for the *cuvette centrale* [6]. Heavy clay soils predominate in Mossaka [1]. The floodplain of Mossaka is located at the confluence of the Congo River and two tributaries, the Sangha River and the Likouala-Mossaka River. The Congo and the Sangha are both clear-water rivers (low sediment load), while the Likouala-Mossaka is a blackwater river [7]

draining a huge area of swamps, marshes and flooded forest on peatland [8]. Because these rivers transport low sediment loads and usually do not overflow in the wet seasons, negligible quantities of sediments are deposited in the floodplain around Mossaka. Flood levels in the plain can sometimes reach up to 150 cm in December during the long rainy season [1]. The area is flooded during the more intense of the two rainy seasons, from September to November. The RFs are normally not flooded, as they are constructed to be high enough to escape flooding and thereby protect the tuberous roots of manioc (*Manihot esculenta*, Euphorbiaceae; also known as cassava), the main crop grown on RFs, from rotting in waterlogged soils.

Past and present agriculture in the study area

Agriculture in the seasonally flooded savannas, marshes and swamps of the Congo basin's *cuvette centrale* is adapted to the region's waterlogged soils. Apart from islands of relatively high ground on old river levees and similar sites, land for cultivation must be reclaimed from water by the building of RFs. Working in an area extending north and west from Mossaka in the 1950's, Sautter[7,9] described the diversity of shapes of RFs, and aerial photographs of one site (Loboko, about 70 km N of Mossaka) showed the extent to which construction of RFs modified landscapes [10]. However, these and other early mentions (see Comptour et al. [1]) give little information on farming practices, and no indication of when RF agriculture in the region may have begun. Since Sautter's time, many of the villages he studied have been abandoned, as people migrated to Mossaka and other settlements close to the river, or to towns outside the region. The current extent of RF agriculture in the *cuvette centrale*, outside of Mossaka, is unknown. In Mossaka today, RF agriculture is declining in importance owing to several factors. Fishing has become more important economically than cultivation of the staple, manioc, much of which is now bought from upland villages endowed with larger areas of well-drained soil. Also, beginning in the 1980's, many people adopted flood-recession agriculture on islands in the Congo River, which is much less labor-intensive than building and maintaining RFs. However, flood-recession agriculture appears to be becoming riskier, with more frequent crop loss in recent years from floods during the short dry season (see Comptour et al. [11] for details).

RF agriculture was once much more widespread than today in the *cuvette centrale*. Vestiges of RFs, and possibly of other human-made earthworks, can be seen in satellite imagery as far north as the headwaters of the Likouala-aux-Herbes (about 300 km N of Mossaka) ([1]; R. Oslisly, pers. comm.). However, although other parts of Africa have contributed noted studies in wetland archaeology[12], no archaeological studies have been conducted in wetland sites in the *cuvette centrale*, and the age of these vestiges is unknown. Interestingly, vestiges of raised fields have also recently been discovered in moist savannas of the lower Ogooué in Gabon (about 900 km W of Mossaka), where dates suggest ages up to 2000 BP[13]. It seems likely that at least some of the Congo RFs are also very old, raising the question

of what crops may have been grown on them before the introduction of manioc from the Americas. Bananas (or plantains) may be a likely candidate. In the early 20th Century, bananas were the main crop grown on RFs at Ngiri, a site in present-day RDC about 325 km NNE of Mossaka [14]. Bananas arrived in Africa at least 2000 years ago, perhaps even earlier[15] and their abundance along the Congo River is historically documented from at least the 16th Century AD [16]. However, only further archaeological research will establish the age of RF agriculture in the *cuvette centrale*, and whether the present-day system is a surviving remnant of an old tradition or a more recent independent invention.

Studied fields and sample collection. Fields N1-N3 were chosen to cover fields of varying age and different phases in the cycle of cultivation. Field N1 (dimensions 4 m diameter x 1.7 m high) was constructed 15-20 years ago. Immediately after construction, it had been planted with stem cuttings of manioc, then left in fallow for at least 10 years before our study. Two weeks before our trench was opened in August 2017, it had been rehabilitated for cultivation: the field and its supplying area (RFSA) were cleared, and a 50-cm layer of vegetation and topsoil was added to the RF surface. Field N2 (dimensions: 7.5 x 7 m x 1.4 m high) was the oldest of the three fields, first constructed more than 40 years ago. After the last fallow period that started about 10 years before our study, in 2016 the field and surrounding area were cleared of vegetation, and new layers of biomass and topsoil were added to the field. It was then planted with manioc. At the time of trench opening, field N2 was thus in its second year of a cycle of cultivation (Fig. 3). Field N3 (dimensions: 4.5 m diameter x 1.3 m high) was constructed 15-20 years ago, had been left fallow for about 10 years, and was in fallow when our trench was opened in August 2017 (Fig. 3).

All three fields (N1-N3) were built by women of the same family using the same techniques. We studied the fields with the permission (and the cooperation) of the owner, who provided information on the age and cultivation stage of each field. In each field we opened a trench using spades to reveal profiles from top to base and across each field. We described profiles and identified standard soil horizons and layers following the guidelines for soil description of the FAO [17]. Following trench opening, we sampled soil from each layer, when these were visible, or at 5-cm depth intervals (total of 220 samples). In each trench, three profiles (X, Y, Z) were sampled (Fig. 2).

Ah horizons (0-10 cm depth) were sampled with an auger in four reference sites for comparison of their properties with those of soils from the RFs. Reference sites were chosen as close as possible to RFs (to minimize natural variation in soil properties) but avoiding intermound sites which were recently used to supply biomass and topsoil for building RFs (i.e., “raised field supplying areas”, RFSA).

We also studied an abandoned field (AF). This field was 2 m broad x 13 m long, and 0.4 m in height. Field AF is located 1 km from fields N1-N3; no abandoned fields occurred closer to these three. Field AF was highly eroded, as nearby active fields were all 1-3 m high. When AF was constructed, and

when it was abandoned, are uncertain, but according to local inhabitants it was abandoned more than 20 years ago.

To better understand bioturbation, 14 separate undisturbed blocks of soils (~ 12cm high x 10cm wide x 4 cm thick) were collected for preparation of thin sections for micromorphological analysis.

Physico-chemical analyses. Colour determination was conducted using a ColorLite Spectrophotometer (sph850m, wavelength 400–700 nm; Katlenburg-Lindau, Germany), measuring spectral reflectance factors on a total of 201 samples (three replicates per sample) that had been air-dried (40°C), then sieved (< 2 mm), and taken from the main profile X of each field. The related software ColorDaTra calculates different colour variables from the reflection spectrum, of which CIELAB colour space is widely applied and increasingly used in soil science [18]. The three variables of this colour space are: L = lightness (0% = black, 100% = white), a = red (+)/green (-), and b = yellow (+)/ blue (-). To visualize the colours, the RGB colour space variables were calculated and the colours displayed as suggested by Sprafke [19]. Total elemental composition was determined by X-ray fluorescence spectroscopy (XRF) in total for six samples (two replicates per RF) representing the C horizon beneath the RFs. For each sample, 4 g of sample milled to powder (grinding machine) and 0.9 g of Licowax C Micropowder were homogenized in an agate mortar for 10 min and pressed into pills with a pressure of about 234 bars. The weathering index was calculated using the Chemical Index of Alteration ($CIA = 100 \times Al_2O_3 / (Al_2O_3 + CaO + Na_2O + K_2O)$) of Nesbitt and Young [20].

Soil extractable mineral and organic P forms were determined on 250 mg sieved fresh soil samples (kept at 4 °C prior to analysis) using 50 ml solution of 0.5 M $NaHCO_3$ (pH 8.5) as chemical extractant [21]. After shaking, the extract was centrifuged for 10 min at 12,830 rpm and then filtered at 45 µm on polyethersulfone (PES) filter to provide two aliquots of the same sample. An aliquot of the $NaHCO_3$ extracts was mineralized in presence of 12N HCl (extract/acid ratio = 1/1) during 16 h at 110 °C [22] to measure inorganic extractable P (P_i). We used the malachite green method to measure inorganic P (P_i) in the mineralized and total extractable P (P_{tex}) in non-mineralized extracts [23] and a spectrophotometer for reading microplates (visible wavelength, ELx808, Dialab, Austria). Organic extractable P (P_o) concentrations were determined as the difference between P_{tex} and P_i. Soil mineral N was extracted with a 1 M KCl solution. NO_3^- and NH_4^+ were determined by continuous flow colorimetry (San^{++} 3000, Skalar, Breda, The Netherlands). Total C and N concentrations were analyzed by dry combustion and gas chromatographic separation with a Flash 2000 CHNS elemental analyzer (Thermo Fisher Scientific, Bremen, Germany). Carbonates were absent; all C was considered organic (C_{org}). To determine pH, the samples were mixed with a 1 M KCl solution and pH was then measured with a glass electrode. The analyses were performed on 194 samples with the exception of colour and pH, for which

only the main profiles X (Fig. 3) were analyzed (59 samples), and of total elemental composition of the clays, for which just one sample per field was measured.

Micromorphology. Fourteen blocks of undisturbed soil taken from the main profile X of fields N2 and N3 were saturated with 100 % ethanol and then gradually impregnated in vacuum by capillarity with a two-component epoxy resin (Araldite 2020). The impregnated blocks were then cut, polished to slices of thickness 30 µm and mounted onto glass slides (8 cm high x 6 cm broad) at the Department of Geosciences, University of Montpellier (Lithopréparation / Broyage - Tri des Minéraux). We examined thin sections from several OM-rich and OM-poor mineral layers from different soil depths (Fig. 3). For all samples, detailed semi-quantitative analyses were made at the petrophysics platform of the Department of Geosciences, University of Montpellier, using a Leica DMLP 2000 microscope in plain-polarized and cross-polarized light (PPL, XPL) following the guidelines of Stoops et al.[24]. The results were arranged and visualized with a colour ramp from white (none) to yellow (few) and red (many) as done by Sprafke[19]. For description of excremental pedofeatures we referred to the guidelines of Bullock et al. [17]. Following the suggestion of Zaiets and Poch [25] for organic horizons, true-colour scans were made with a high-resolution Epson scanner to better explore boundaries and macropores.

SI Figures

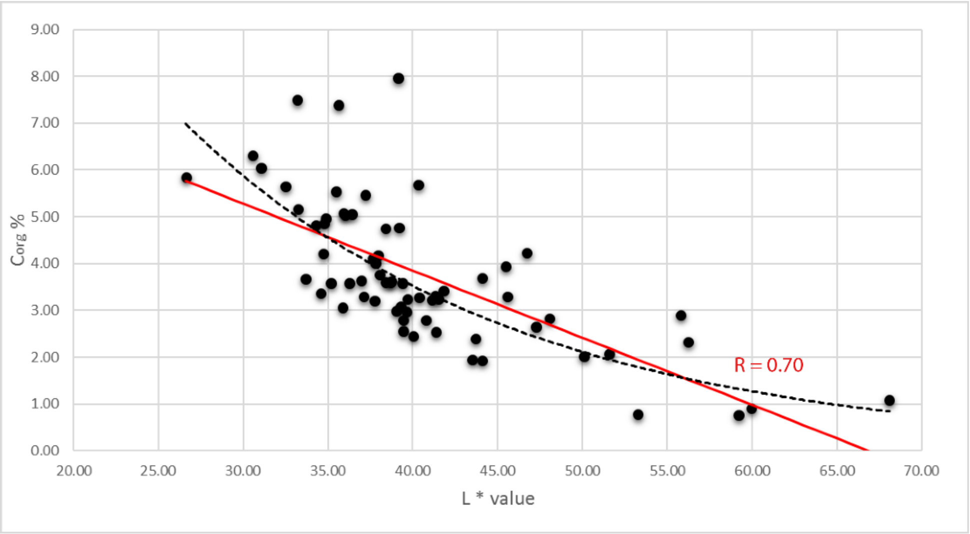

Figure S1 Correlation between C_{org} % and L*-value (Lightness index). The red line represents the linear trend line with R = -0.703; p = 3.3 e-11. The black dotted curve indicates the asymptotic trend line.

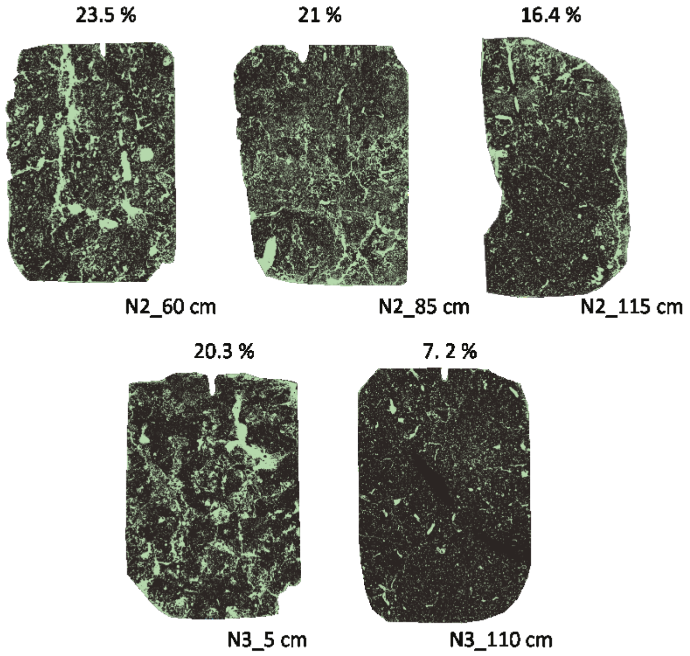

Figure S2 Examples for visualized differences in amount of porosity (green) calculated using image analysis tools (ArcGIS) at different depths (labelled at the bottom) of profile X in fields N2 and N3 (compare Table S4).

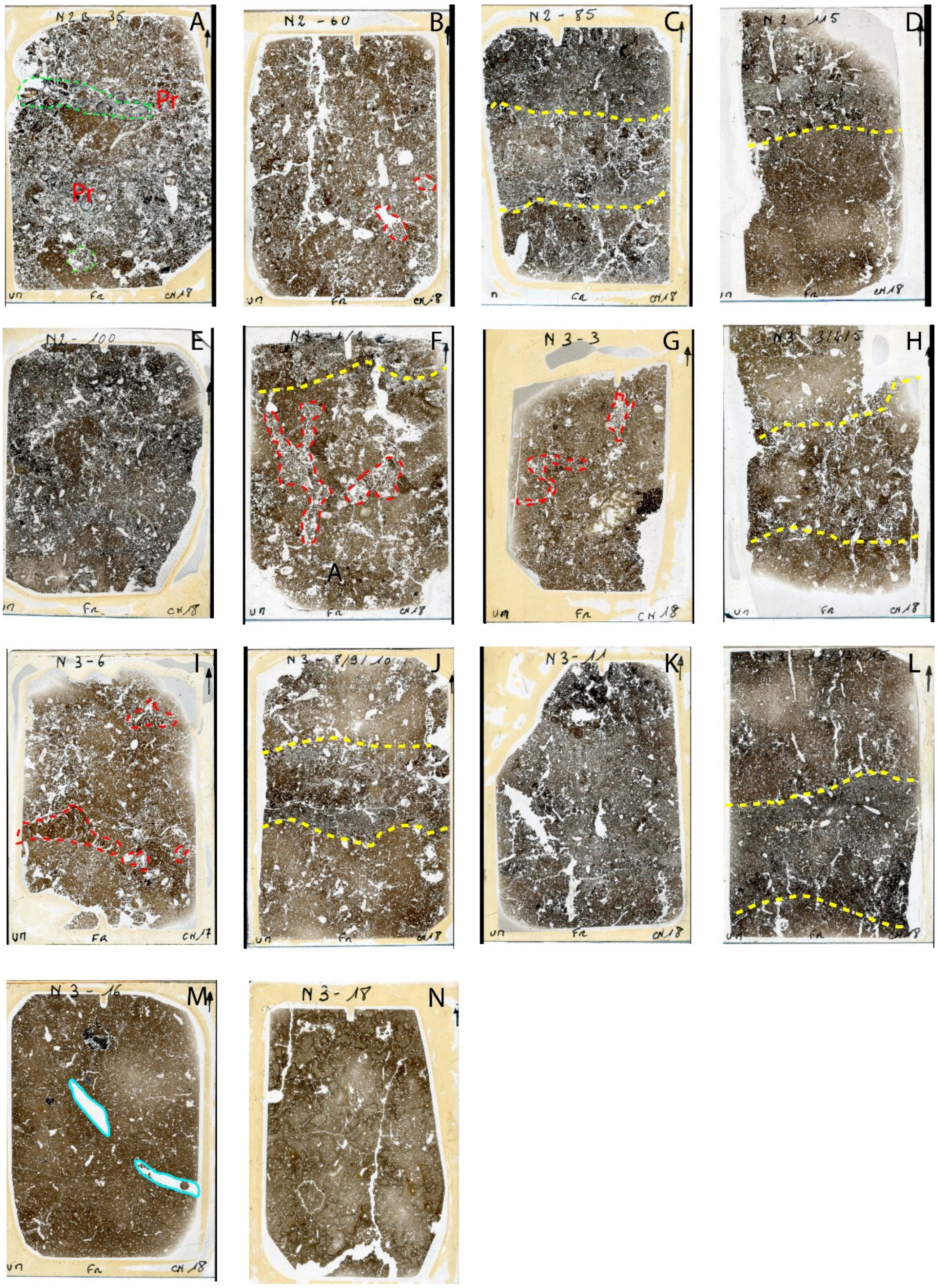

Figure S3. A-N: Scanned thin sections from different depths of profile X in fields N2 and N3; width of slides 60 mm. A-E: field N2. A: 35 cm; mixed organic-mineral layer with thin layer (dashed green line) of poorly decomposed and charred plant remains (Pr). B: 60 cm; highly bioturbated organic-mineral layer, voids and channels partly infilled with OM-rich excrements (red dashed lines). C: 85 cm; vertical cut through three layers, partly mixed but with boundaries still visible (yellow dashed lines). Decomposed vegetation layer (top), OM-rich mineral layer (middle), and decomposed vegetation layer (bottom). D: 115 cm; vertical cut through two layers, separated by yellow dashed lines: Decomposed vegetation (top), OM-rich mineral layer with relatively low porosity (bottom). E: 100 cm; mixed organic-mineral layer, very OM-rich. F-M: field N3. F: 5 cm; vertical cut through two layers separated by yellow dashed lines: OM-rich layer representing Ah of the field (top), well-mixed organic-mineral layer (bottom). Most of the voids are infilled with OM-rich faunal excrement. F-N: field N3. G: 10 cm; mixed mineral layer, voids infilled with OM-rich faunal excrements. Vertical cut though three layers separated by yellow dashed lines. H: 12-20 cm; same layers as G at 10 cm (top), decomposed vegetation layer (middle), organic-rich mineral layer (bottom). I: 30 cm;

well-mixed, OM-rich mineral layer, voids infilled with faunal excrement. J: 45-53 cm; vertical cut through three layers separated by yellow dashed lines: organic- mineral layer (top), decomposed vegetation layer (middle), mineral layer (bottom). K: 65 cm; decomposed vegetation layer. L: 83-95 cm; vertical cut though three layers separated by yellow dashed lines: very OM-rich mineral layer (top), decomposed vegetation layer, void (red dashed line) partly infilled with excrements of invertebrates. M: 110 cm; mineral layer, very dense, low porosity, stained by organic matter. N: 135 cm: mineral layer, very dense, low porosity, stained by organic matter. Dashed yellow lines (C, D, F, H, J) are the approximate boundaries between organic and mineral layers. Dashed red lines (B, F, G, I) are the contours of some of the mesofaunal and vegetation-derived channels (infilled with OM-rich excrements). The areas outlined by a blue line (in L) indicate artefacts of the preparation of the thin section. The green dashed line (A) indicates a layer with poorly decomposed plant remains, which are indicated in red (Pr). Semi-quantitative analysis of microstructure and pedofeatures is given in Table S4.

SI Tables

Table S1 C_{org} content, spectrophotometrically measured CIELAB colour space values and converted RGB values, for the main profile (X) in each of the four studied fields.

Depth	C	L*	a*	b*	R	G	B	Sum L*a*b*
cm:	g kg^{-1}							
Field N1								
0	33	45.6	2.0	6.7	115.87	106.52	96.09	54.25
10	36	39.4	1.9	5.8	99.96	91.51	82.74	47.19
17	32	41.1	2.2	6.4	104.95	95.48	85.90	49.74
30	26	47.3	2.1	7.4	120.78	110.61	98.94	56.80
40	33	40.4	2.1	6.1	102.65	93.73	84.62	48.51
47	34	41.8	2.3	6.6	106.88	97.09	87.20	50.70
50	36	38.4	2.0	5.8	97.40	89.06	80.48	46.09
57	48	38.4	2.4	5.3	97.88	88.89	81.21	46.11
62	36	38.7	2.2	5.0	98.19	89.80	82.55	45.90
70	38	38.0	2.2	4.7	96.26	88.20	81.34	44.91
85	60	31.1	1.7	3.2	77.99	72.14	67.68	35.97
93	33	41.3	2.2	5.4	104.77	95.95	87.92	48.92
95	50	34.8	2.0	3.7	87.73	80.75	75.49	40.58
105	28	39.5	2.4	5.2	100.45	91.41	83.88	47.09
107	52	33.2	1.5	3.1	82.79	77.28	72.74	37.90
110	30	39.6	2.4	5.4	101.06	91.85	84.02	47.48
120	48	34.3	1.7	3.6	85.85	79.52	74.26	39.64
124	19	44.1	2.5	6.0	112.60	102.61	93.68	52.61
150	36	35.1	2.2	4.3	89.03	81.35	75.23	41.63
160	75	33.2	1.2	2.4	81.64	77.30	73.64	36.80
165	34	34.6	2.2	4.2	87.65	79.96	74.12	40.96
170	9	60.0	2.1	7.9	153.94	142.87	129.73	69.90
Field N2								
0	28	48.0	1.8	5.7	121.18	112.82	103.76	55.52
10	21	51.6	2.2	6.8	131.69	121.42	110.56	60.65
20	55	37.2	2.0	4.3	93.68	86.32	80.02	43.48
28	50	36.0	1.5	3.4	89.47	83.81	78.66	40.90
29	32	41.5	2.1	4.7	104.62	96.60	89.48	48.31
37	42	38.0	1.6	3.8	94.67	88.32	82.61	43.40
40	32	39.7	2.1	4.6	100.11	92.11	85.34	46.39
44	32	37.7	1.8	4.1	94.56	87.67	81.51	43.66
53	25	41.4	2.3	5.4	105.16	96.13	88.18	49.09
59	36	37.0	1.9	3.9	92.81	85.83	80.20	42.77
70	41	37.7	1.8	3.5	94.02	87.52	82.42	42.95
80	40	37.8	1.7	3.1	93.80	87.91	83.26	42.53
85	48	39.2	1.8	3.6	97.83	91.19	85.83	44.61
88	55	35.5	1.5	2.5	86.16	80.71	77.33	39.47
95	51	35.9	1.4	2.6	88.48	83.68	79.77	39.83
105	51	36.4	1.4	2.9	89.91	84.78	80.28	40.71
113	57	32.5	1.4	2.2	80.18	75.71	72.49	36.06
116	26	39.4	2.2	4.9	99.77	91.45	84.23	46.51
130	8	59.2	2.5	8.6	153.07	140.65	126.54	70.26

Table S1 continued

Depth	C	L*	a*	b*	R	G	B	Sum L*a*b*
cm:	g kg^{-1}							
**Field N3**								
0	74	35.6	1.3	2.8	87.88	82.93	78.66	39.74
1	36	38.7	1.6	4.0	96.55	90.13	84.12	44.28
7	20	50.1	2.2	6.5	127.48	117.62	107.30	58.77
17	28	40.8	2.3	5.0	103.36	94.69	87.33	48.07
22	25	40.0	2.1	5.0	101.22	92.91	85.55	47.13
30	30	39.0	2.2	4.6	98.64	90.58	83.91	45.79
42	49	34.8	1.8	3.3	86.90	80.78	76.06	39.82
48	31	39.3	2.2	4.8	99.42	91.14	84.05	46.31
53	42	34.7	1.7	3.2	86.56	80.66	76.02	39.58
60	33	37.1	2.1	4.3	93.64	85.94	79.80	43.50
61	63	30.5	1.4	2.2	75.65	71.10	67.97	34.15
73	36	36.2	2.0	3.6	90.95	84.08	78.83	41.86
84	31	35.9	2.2	3.7	90.43	83.13	77.98	41.72
94	59	26.6	1.0	1.1	65.34	62.44	60.84	28.75
98	37	33.7	1.7	3.1	84.00	78.16	73.69	38.46
104	20	43.5	2.6	5.8	111.08	101.05	92.54	51.87
117	24	43.7	2.3	5.1	110.64	101.81	94.24	51.04
130	8	53.2	2.5	8.1	137.30	125.34	112.50	63.90
**Field AF**								
0.00	80	39.2	1.8	6.1	99.31	90.97	81.70	47.11
3.00	37	44.1	2.0	6.5	112.05	102.83	92.84	52.59
5.00	29	55.8	2.4	7.8	143.38	131.92	119.39	65.91
10.00	57	40.3	1.5	4.8	100.79	93.99	86.56	46.59
17.00	39	45.5	2.2	6.1	115.57	106.18	96.81	53.76
28.00	42	46.7	2.2	5.5	118.20	109.24	100.80	54.33
30.00	23	56.2	2.3	7.3	144.08	133.13	121.25	65.81
35.00	11	68.1	3.3	12.3	180.75	163.34	142.82	83.68

Table S2 Selected elements of the clay samples taken at the base of the main profile X in fields N1, N2, N3 and calculation of CIA.

Sample Name	Depth	SiO_2	Al_2O_3	Na_2O	K_2O	CaO	MgO	P_2O_5	P	CIA index
		%	%		%	%	%	%	mg kg^{-1}	
N1	180 cm	58.24	21.92	< 0,0014	0.93	< 0,0014	0.37	0.09	403.55	95.95
N2	135 cm	59.54	20.31	< 0,0014	0.89	< 0,0014	0.33	0.09	401.45	95.79
N3	140 cm	58.93	20.48	< 0,0014	0.93	< 0,0014	0.36	0.09	397.4	95.65

Table S3 Chemical analysis of the three profiles X, Y, and Z of each of the four fields (N1, N2, N3 and AF) and of reference topsoils.

Depth	pH	C_{org}	N	C/N	P_o	P_i	NH_4	NO_3
cm:		g kg^{-1}	g kg^{-1}		mg kg^{-1}	mg kg^{-1}	mg kg^{-1}	mg kg^{-1}
Field N1								
N1X								
0	3.7	33	2	14.4	10.0	5.5	15.4	0.4
10	3.7	36	2	14.4	10.8	4.4	20.7	0.4
17	3.6	32	2	14.1	9.8	7.5	28.2	0.7
30	3.6	26	2	13.2	16.1	5.9	25.7	0.1
40	3.6	33	2	13.9	12.7	6.3	15.3	0.5
47	3.6	34	2	13.9	7.1	2.3	7.9	0.0
50	3.7	36	3	14.3	13.6	15.4	19.4	0.2
57	3.5	48	3	15.9	13.3	3.4	12.4	0.0
62	3.6	36	2	15.1	13.6	4.5	13.0	0.2
70	3.6	38	2	15.6	13.2	2.9	4.5	0.1
85	3.6	60	3	18.2	14.3	2.7	4.8	0.2
93	3.6	33	2	16.9	9.6	2.1	10.8	0.1
95	3.6	50	3	17.1	10.3	2.2	7.2	0.4
105	3.6	28	2	14.9	12.6	2.7	9.2	5.9
107	3.6	52	3	18.0	17.0	3.4	6.7	9.2
110	3.6	30	2	15.5	15.1	2.1	10.0	4.3
120	3.5	48	3	17.1	11.8	2.2	6.1	6.0
124	3.5	19	1	14.3	6.4	1.3	8.8	1.7
150	3.6	36	2	16.8	11.6	2.0	4.2	5.5
160	3.6	75	4	19.7	16.2	2.6	3.9	10.3
165	3.5	34	2	16.1	8.8	1.9	9.4	1.5
N1 Y								
0	-	48	3	15.1	6.4	22.2	23.9	9.1
10	-	31	2	14.6	4.1	15.5	15.1	0.6
17	-	56	3	17.3	12.1	25.6	30.2	0.7
30	-	41	3	14.3	4.6	15.3	12.6	0.1
40	-	51	3	15.2	8.9	28.8	19.0	0.2
47	-	20	2	12.4	2.7	7.1	1.8	0.1
50	-	90	5	19.5	13.6	35.8	17.0	8.9
57	-	74	4	18.6	11.6	31.6	22.5	3.0
62	-	37	2	16.4	4.6	17.9	13.5	0.1
70	-	45	3	17.3	3.3	16.8	8.9	0.1
85	-	64	3	19.5	3.0	18.9	7.6	0.1
93	-	33	2	17.6	1.9	28.4	2.6	0.1
95	-	52	3	17.7	2.6	19.2	4.0	2.8
106	-	52	3	17.6	3.0	18.7	1.3	0.1
107	-	50	3	18.6	-	-	6.7	5.2
110	-	38	2	17.3	3.0	18.7	6.0	5.3
120	-	48	3	18.3	1.3	12.0	15.1	2.6
124	-	18	1	14.6	1.1	10.2	7.2	0.5
150	-	46	2	18.8	1.6	20.0	3.4	7.5
160	-	78	4	20.6	2.3	25.9	6.4	8.7
165	-	32	2	17.4	1.5	20.5	3.8	3.5
170	-	10	1	10.9	0.6	10.6	5.6	0.2

Table S3 continued

Depth	pH	C_{org}	N	C/N	P_o	P_i	NH_4	NO_3
cm:		g kg^{-1}	g kg^{-1}		mg kg^{-1}	mg kg^{-1}	mg kg^{-1}	mg kg^{-1}
Field N1								
				N1Z				
0	-	35	2	15.2	21.7	4.1	8.5	2.4
10	-	32	2	14.7	15.7	6.3	10.5	0.3
17.	-	47	3	15.7	23.9	8.9	17.7	0.8
30	-	31	2	13.4	16.8	3.9	8.6	0.2
40	-	36	2	15.1	18.7	4.4	12.5	0.4
47	-	42	3	15.2	16.4	17.7	25.9	1.7
50	-	38	2	16.4	27.9	4.5	34.1	1.6
57	-	68	4	18.2	28.0	9.3	18.5	0.4
62	-	34	2	15.3	19.8	3.0	20.8	0.2
70	-	43	2	17.6	19.2	1.9	6.4	0.2
85	-	59	3	18.5	21.2	2.3	6.3	0.5
93	-	35	2	16.6	17.0	1.4	4.0	0.2
95	-	67	4	18.4	26.0	2.8	6.7	1.3
106	-	34	2	16.9	17.5	1.4	3.9	0.5
107	-	64	3	20.5	24.1	2.5	5.5	3.2
110	-	28	2	16.4	10.0	1.7	5.0	1.1
120	-	55	3	18.3	22.6	2.0	5.3	7.7
124	-	23	1	15.9	8.2	-0.9	9.9	1.4
150	-	67	3	20.3	18.0	2.5	4.2	11.5
160	-	82	4	21.8	23.4	3.1	4.1	11.7
165	-	-	-	-	17.2	1.3	3.8	6.5
170	-	9	1	10.6	5.8	0.4	5.1	0.5
Field N2								
				N2X				
0	3.7	28	2	13.4	10.6	13.6	12.4	7.8
10	3.5	21	2	11.6	2.8	2.8	0.0	0.3
20	3.5	55	3	16.5	16.5	10.1	23.4	9.8
28	3.5	50	3	17.1	16.5	17.0	17.4	37.3
29	3.5	32	2	13.8	15.0	4.9	7.1	23.4
37	3.5	42	3	16.0	13.5	10.3	22.1	13.8
40	3.6	32	2	14.8	8.1	2.7	15.8	0.3
44	3.6	32	2	15.7	10.4	2.9	11.5	0.7
53	3.6	25	2	14.9	7.4	1.8	8.3	0.7
59	3.6	36	2	16.7	10.1	2.3	5.5	3.4
70	3.6	41	2	17.5	13.8	2.9	7.5	2.7
80	3.6	40	2	17.5	13.6	2.6	7.5	2.4
85	3.6	48	3	18.9	15.7	3.0	10.6	2.1
88	3.6	55	3	20.5	12.4	2.7	7.9	3.3
95	3.5	51	3	20.0	16.7	3.2	7.5	3.2
105	3.6	51	2	20.4	14.3	3.1	8.0	2.7
113	3.6	57	3	19.8	13.9	3.0	5.1	3.8
116	3.5	26	2	16.4	5.2	0.9	6.8	0.2
130	3.4	8	1	10.8	1.9	0.4	4.5	0.0

Table S3 continued

Depth	pH	C_{org}	N	C/N	P_o	P_i	NH_4	NO_3
cm:		g kg^{-1}	g kg^{-1}		mg kg^{-1}	mg kg^{-1}	mg kg^{-1}	mg kg^{-1}
Field N2								
				N2Y				
-15	-	30	2	15.2	12.1	6.1	18.9	8.2
-10	-	27	2	12.8	10.2	3.5	31.7	0.2
-5	-	24	2	13.0	12.5	4.2	10.9	0.2
0	-	32	2	13.4	15.3	4.7	19.8	0.1
5	-	31	2	15.1	16.7	4.9	20.8	1.7
10	-	36	2	16.9	15.6	5.6	22.8	3.7
15	-	18	1	12.5	10.1	2.9	23.8	0.5
20	-	16	1	12.7	9.5	2.6	13.0	0.1
25	-	24	2	13.1	12.1	4.9	23.6	1.8
30	-	28	2	14.3	14.7	3.5	18.2	1.3
35	-	36	2	15.9	13.7	2.6	11.4	1.2
40	-	30	2	16.1	10.5	1.8	10.8	0.3
45	-	27	2	15.9	16.6	0.1	9.7	0.2
50	-	32	2	16.2	15.8	2.7	9.9	0.3
60	-	29	2	16.1	15.3	2.3	6.8	0.9
65	-	33	2	16.7	7.5	2.4	7.4	2.2
70	-	45	2	18.2	18.2	2.8	9.4	2.5
75	-	49	3	19.1	26.4	3.4	8.3	3.5
80	-	55	3	19.4	23.3	2.6	8.3	3.3
58	-	43	2	18.7	20.5	1.8	5.8	4.5
90	-	44	2	18.4	21.2	1.8	7.6	2.6
95	-	41	2	18.2	19.5	1.8	12.2	1.4
100	-	56	3	20.2	29.1	1.9	7.0	4.2
105	-	33	2	16.5	16.7	1.6	7.1	1.5
110	-	18	1	15.5	10.5	0.9	4.0	0.5
115	-	08	1	11.0	5.6	0.3	2.8	0.2
120	-	0.	1	10.6	7.4	0.6	4.1	0.2
				N2Z				
-10	-	28	2	14.6	18.0	8.6	14.7	5.2
-5	-	26	2	15.0	1.5	3.1	17.4	1.7
0	-	22	2	13.2	10.2	3.7	14.8	1.2
5	-	27	2	13.7	12.3	3.7	14.4	0.1
10	-	24	2	15.1	9.3	4.4	9.5	0.3
15	-	51	3	16.9	22.9	23.3	37.0	1.6
20	-	23	2	14.2	11.6	4.6	17.3	1.3
25	-	16	1	12.5	12.2	2.2	9.1	2.3
30	-	15	1	12.3	10.0	2.5	15.7	0.6
35	-	16	1	12.7	11.3	2.3	12.0	0.5
40	-	20	1	13.9	10.4	5.7	8.3	2.0
45	-	22	2	14.6	14.0	1.6	11.2	1.9
50	-	17	1	13.7	10.9	1.4	9.4	1.2
60	-	20	1	14.4	14.3	2.2	15.1	3.7
65	-	28	2	15.7	19.8	2.3	12.8	8.3
70	-	30	2	16.3	12.7	1.8	7.4	4.7
75	-	28	2	16.2	13.2	2.1	8.4	6.9
80	-	29	2	15.8	13.8	1.8	4.0	7.6
58	-	31	2	17.1	17.8	1.8	3.9	11.2
90	-	33	2	17.1	15.8	1.7	5.7	6.1
95	-	30	2	16.8	13.8	1.7	8.3	2.1
100	-	30	2	16.5	8.4	2.0	12.3	0.9
105	-	19	1	15.3	9.9	2.1	5.6	1.0
110	-	20	1	14.5	6.3	1.6	7.3	0.9
115	-	21	1	15.1	10.6	2.4	9.1	1.1
110	-	8	1	11.1	5.1	0.6	4.1	0.1

Table S3 continued

Depth	pH	C_{org}	N	C/N	P_{org}	P_i	NH_4	NO_3
cm:		g kg^{-1}	g kg^{-1}		mg kg^{-1}	mg kg^{-1}	mg kg^{-1}	mg kg^{-1}
Field N3								
N3X								
0	3.6	74	4	21.7	20.1	15.2	27.9	6.9
1	3.5	36	2	15.1	10.6	3.5	6.2	0.1
7	3.6	20	1	14.2	6.5	1.7	5.8	0.0
17	3.5	28	2	14.6	10.2	3.0	8.3	0.0
22	3.6	25	2	15.9	7.8	1.9	5.9	0.0
30	3.6	30	2	16.1	9.7	2.6	5.0	0.0
42	3.5	49	3	18.2	13.3	2.4	4.6	1.3
48	3.6	31	2	17.0	13.5	2.5	7.3	0.5
53	3.5	42	3	19.9	18.5	2.9	5.3	2.7
60	3.5	33	2	16.9	13.3	2.6	7.5	1.2
61	3.5	63	3	21.2	17.0	1.8	3.7	2.8
73	3.5	36	2	17.3	15.6	2.4	5.7	0.7
84	3.5	31	2	17.2	13.0	2.0	8.8	0.1
94	3.5	59	3	20.2	14.1	2.7	5.3	1.1
98	3.5	37	2	18.7	10.5	2.1	3.9	0.0
104	3.4	20	1	17.6	4.3	1.3	1.8	0.0
117	-	24	1	18.8	6.3	1.4	3.8	0.0
130	-	8	1	11.0	1.0	0.5	3.0	0.0
N3Y								
0	-	58	3	18.0	9.8	7.9	10.5	0.0
1	-	43	2	17.3	6.7	8.3	12.8	0.0
4	-	24	2	13.9	5.5	4.4	2.9	0.0
28	-	33	2	17.4	5.5	3.5	3.8	0.0
28b	-	22	1	15.0	4.6	2.0	2.8	0.0
40	-	43	3	17.1	8.2	3.0	3.7	0.0
51	-	48	3	19.0	8.5	2.9	7.0	0.1
56	-	28	2	16.3	12.4	3.8	6.1	1.5
56b	-	27	2	16.8	6.0	2.4	2.4	0.0
61	-	65	3	21.3	13.4	4.2	5.1	3.1
69	-	38	2	18.6	10.0	3.2	5.9	0.1
79	-	33	2	16.8	8.6	3.4	7.5	0.3
90	-	49	3	18.1	12.7	4.1	3.6	0.0
95	-	33	2	20.3	5.5	1.9	1.9	0.0
100	-	14	1	14.5	2.7	1.1	3.3	0.0
N3Z								
0	-	31	2	15.9	4.1	3.4	5.5	0.0
7	-	21	1	14.3	14.0	1.8	2.8	0.0
36	-	34	2	17.5	5.3	3.4	6.6	0.0
40	-	22	1	14.7	7.8	2.6	3.9	0.0
55	-	49	3	18.0	10.6	4.1	5.4	1.0
59	-	25	2	15.4	12.9	1.5	3.6	0.0
80	-	40	2	17.2	9.8	4.0	3.6	0.0
84	-	35	2	16.3	11.5	3.3	3.4	0.0
87	-	62	3	20.1	11.7	3.9	3.8	0.5
94	-	36	2	17.5	7.1	3.3	4.3	0.0
100	-	17	1	14.4	-0.4	1.7	3.1	0.0
105	-	8	1	9.8	0.4	0.9	4.5	0.0
AF (only profile X was done for this field)								
0	-	80	5	15.2	22.2	5.9	29.4	0.0
3	-	37	3	14.3	0.6	5.8	15.8	0.0
5	-	29	2	13.6	3.1	3.8	11.9	0.0
10	-	57	3	16.6	7.6	4.9	9.3	0.16
17	-	39	2	16.4	4.4	4.2	9.4	0.0
28	-	42	3	16.2	6.5	2.8	4.9	0.2
30	-	23	2	14.1	2.3	2.7	5.7	0.0
35.00	-	11	1	10.48	-0.15	0.60	3.21	0.00

Table S3 continued

Sample No	pH	C_{org}	N	C/N	P_{org}	P_i	NH_4	NO_3
		g kg^{-1}	g kg^{-1}		mg kg^{-1}	mg kg^{-1}	mg kg^{-1}	mg kg^{-1}
Reference topsoils (0-10 cm depth)								
589 A	3.8	48	3	15.1	17.8	4.2	8.3	0.0
589 B	3.7	27	2	13.1	10.8	4.9	8.0	2.4
590	3.8	29	2	14.0	5.0	1.4	2.4	0.0
591	3.9	33	2	14.0	9.4	3.2	2.2	0.0
Mean	*3.8*	*34*	*2*	*14.0*	*10.7*	*3.4*	*5.3*	*0.6*

Table S4 Semi-quantitative analysis of micromorphological characteristics. Colours show the relative frequencies of the respective features (0-5).

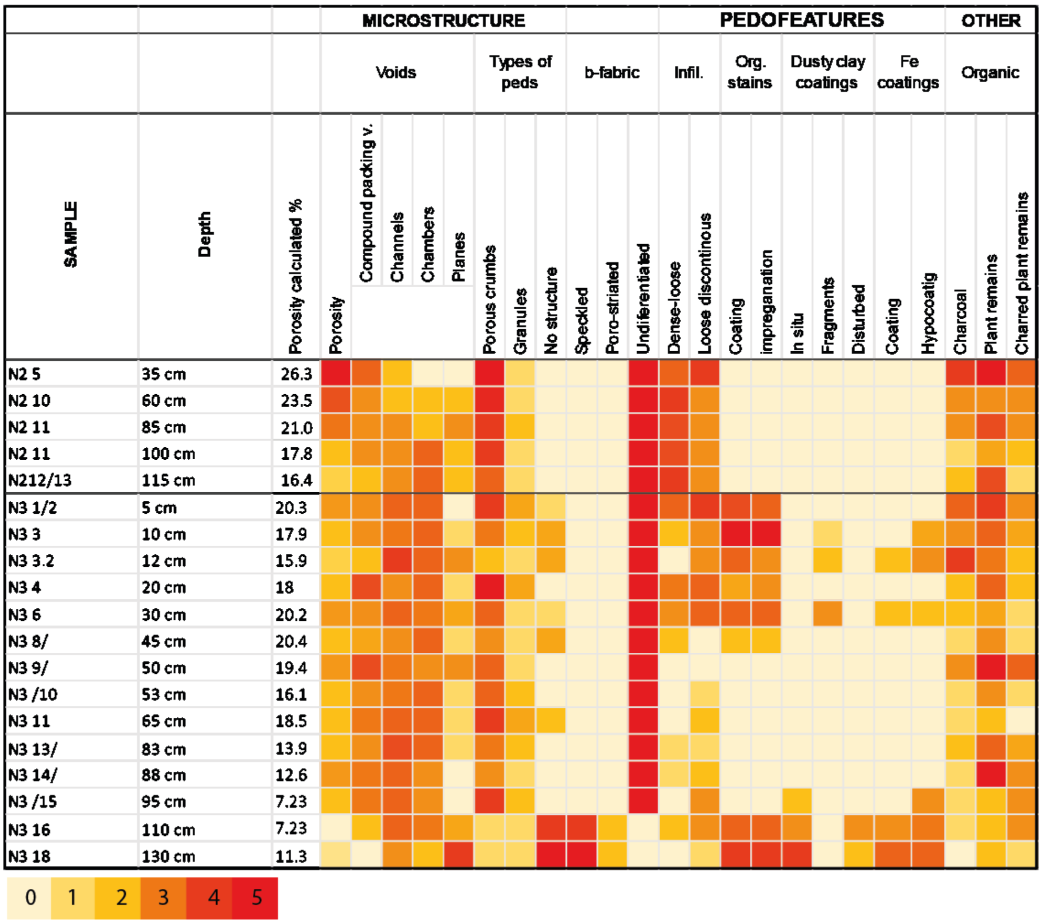

SI References

1. Comptour, M., Caillon, S., Rodrigues, L. & McKey, D. Wetland raised-field agriculture and its contribution to sustainability: ethnoecology of a present-day African system and questions about pre-Columbian systems in the American tropics. *Sustainability* **10**, 3120 (2018).
2. Comptour, M. *Entre pêche, agriculture, et commerce, jouer avec la variabilité écologique et sociale : Dynamique d'un système social-écologique dans les plaines inondables du fleuve Congo*. (PhD thesis, University of Montpellier, 2017).
3. Campbell, D. *The Congo River Basin*. (The World's Largest Wetlands: Ecology and Conservation. Cambridge (United Kingdom): Cambridge University Press, 2005).
4. Samba, G. & Nganga, D. Rainfall variability in Congo–Brazzaville: 1932–2007. *Int. J. Climatol.* **32**, 854–873 (2012).

5. Lee, H., Yuan, T., Jung, H. C. & Beighley, E. Mapping wetland water depths over the central Congo Basin using PALSAR ScanSAR, Envisat altimetry, and MODIS VCF data. *Remote Sens. Environ.* **159**, 70–79 (2015).
6. Yoka, J., Loumeto, J. J., Vouidibio, J., Amiaud, B. & Epron, D. Influence du sol sur la répartition et la production de phytomasse de savanes de la Cuvette congolaise (République du Congo). *Géo-Eco-Trop Liège* **34**, 63–74 (2010).
7. Sautter, G. *La Cuvette Congolaise : Monographie Régionale des Bassins de la Likouala-Mossaka, de l'Alima et de la Nkéni*. (Ministère de la coopération, 1962).
8. Dargie, G. C. *et al.* Age, extent and carbon storage of the central Congo Basin peatland complex. *Nature* **542**, 86–90 (2017).
9. Sautter, G. *De l'Atlantique au Fleuve Congo: Une Géographie du Sous-Peuplement*. (République du Congo, République Gabonaise; Mouton et Cie: La Hay, 1966).
10. Auger, A. Loboko: Exemple de terroir conquis sur l'eau. *Photo-Interprétation* **6**, 22–28 (1967).
11. Comptour, M. *et al.* Agricultural innovation and environmental change on the floodplains of the Congo River. *Geogr. J.* **186**, 16–30 (2020).
12. Menotti, F. *Wetland Archaeology and beyond: Theory and Practice*. (Oxford University Press, 2012).
13. Oslisly, R. Découvertes d'anciennes traces de structures agraires dans les savanes humides du Bas Ogooué. In: J.P. Van de weghe & T. Stévart (eds.), *Le Delta de l'Ogooué.* Agence Nationale des Parcs Nationaux, Libreville, Gabon, pp. 128-129. (2017).
14. Mumbanza mwa Bawele J & Nyakombi Ensobato. La production alimentaire dans les marais de la Haute-Ngiri du XIXe siècle à nos jours. *Afr. Econ. Hist.* 130–139 (1979).
15. Lejju, B. J., Robertshaw, P. & Taylor, D. Africa's earliest bananas? *J. Archaeol. Sci.* **33**, 102–113 (2006).
16. Wigboldus, J. S. The spread of crops into sub-equatorial Africa during the Early Iron Age: a 'minimalist'view based primarily on documentary evidence from the Indian Ocean side. *Azania Archaeol. Res. Afr.* **29**, 121–129 (1994).
17. IUSS Working Group WRB. *World Reference Base for Soil Resources: International soil classification system for naming soils and creating legends for soil maps*. (FAO, 2014).
18. Viscarra Rossel, R. A., Minasny, B., Roudier, P. & McBratney, A. B. Colour space models for soil science. *Geoderma* **133**, 320–337 (2006).
19. Sprafke, T. *Löss in Niederösterreich - Archiv quartärer Klima- und Landschaftsveränderungen*. (PhD thesis,Würzburg University Press, 2016).
20. Nesbitt, H. W. & Young, G. M. Early Proterozoic climates and plate motions inferred from major element chemistry of lutites. *Nature* **299**, 715–717 (1982).
21. Olsen, S. R. *Estimation of Available Phosphorus in Soils by Extraction with Sodium Bicarbonate* (United States Department Of Agriculture; Washington, 1954).
22. Ali, M. A., Louche, J., Legname, E., Duchemin, M. & Plassard, C. Pinus pinaster seedlings and their fungal symbionts show high plasticity in phosphorus acquisition in acidic soils. *Tree Physiol.* **29**, 1587–1597 (2009).
23. Ohno, T. & Zibilske, L. M. Determination of low concentrations of phosphorus in soil extracts using malachite green. *Soil Sci. Soc. Am. J.* **55**, 892–895 (1991).
24. Stoops, G., Marcelino, V. & Mees, F. *Interpretation of Micromorphological Features of Soils and Regoliths*. (Elsevier, 2010).
25. Zaiets, O. & Poch, R. M. Micromorphology of organic matter and humus in Mediterranean mountain soils. *Geoderma* **272**, 83–92 (2016).
